# Exploring Paediatric Jaw Osteosarcoma: A Detailed Case Report of a 12-Year-Old Girl and Review of the Literature

**DOI:** 10.1155/crid/7428891

**Published:** 2025-04-22

**Authors:** Jessica Simpson, Moegamat Sallies, Amir H. Afrogheh

**Affiliations:** ^1^Department of Craniofacial Biology, Pathology & Radiology, Faculty of Dentistry, University of The Western Cape, Cape Town, South Africa; ^2^Department of Oral and Maxillofacial Surgery, Faculty of Dentistry, University of The Western Cape, Cape Town, South Africa; ^3^Division of Anatomical Pathology, Stellenbosch University, Cape Town, South Africa; ^4^Department of Oral and Maxillofacial Pathology, National Health Laboratory Service, University of the Western Cape, Cape Town, South Africa; ^5^National Health Laboratory Service, Tygerberg Hospital, Cape Town, South Africa

**Keywords:** case report, malignancy, oral pathology, osteosarcoma, paediatric dentistry

## Abstract

Osteosarcoma, the most common primary bone malignancy, rarely affects the jaw, representing only 6%–10% of cases. Jaw osteosarcoma typically occurs in individuals between the ages of 30 and 50 years and is uncommon in children. It often involves the mandible, especially in males, presenting with swelling, tooth mobility, and paraesthesia rather than pain. We present a rare case of jaw osteosarcoma in a 12-year-old girl who exhibited paraesthesia and a significant mandibular mass. Imaging demonstrated extensive mandibular involvement with Codman's triangle formation, and a biopsy confirmed the diagnosis of conventional osteosarcoma. The treatment approach included a hemimandibulectomy, followed by temporary reconstruction using a titanium plate and silastic spacer. This case underscores the importance of comprehensive evaluation and multidisciplinary management in diagnosing and treating osteosarcoma of the jaws in children. A review of 12 similar cases highlights the variability in presentation and treatment outcomes, emphasizing the need for individualized treatment plans to optimize patient prognosis.

## 1. Introduction

Although osteosarcoma is the most common primary malignancy of bone, only 6%–10% [1] of these lesions are localized to the jaws. When osteosarcoma of the jaw (OSJ) does occur, the affected individuals are usually between the ages of 30 and 50 years [2]. In children, the jaw is an uncommon location for osteosarcoma development.

Osteosarcoma of the axial skeleton typically affects the metaphysis of long bones during periods of skeletal development in children and adolescents, often in the second decade of life [3, 4]. However, OSJ usually occurs in the mandible, peaking one or two decades after adolescence. Males are more commonly affected by OSJ than females, with most tumours located in the mandibular molar region [1, 5].

Unlike osteosarcomas of long bones, OSJ typically presents with swelling rather than pain [3]. Patients may also report a rapidly growing soft tissue mass, tooth mobility, ulceration, nasal obstruction, and paraesthesia [6–8]. The mandibular body is the most frequently affected area, followed by the angle, symphysis, and ascending ramus. In the maxilla, the posterior alveolar process and maxillary sinus are commonly involved [9]. Osteosarcoma in the maxilla typically peaks in the 5th decade of life, while mandibular osteosarcoma occurs with similar frequency in both middle-age and older individuals [10].

Several case reports have documented paediatric OSJ, emphasizing its rarity and diagnostic challenges. Among them, cases reported by Nirmala et al. [11] and Madiraju [12] involved female patients with conventional osteosarcoma of the mandible, contributing to the understanding of its clinical presentation and management. However, certain radiographic features remain infrequently described despite these reports.

To the best of our research, no previously reported paediatric cases have demonstrated the presence of Codman's triangle, making our case unique. This report presents a rare instance of OSJ in a child with no known contributing factors. By contextualizing our findings within the existing literature, this review aims to enhance the understanding of its diagnosis and treatment.

## 2. Case Report

**Figure 1:**
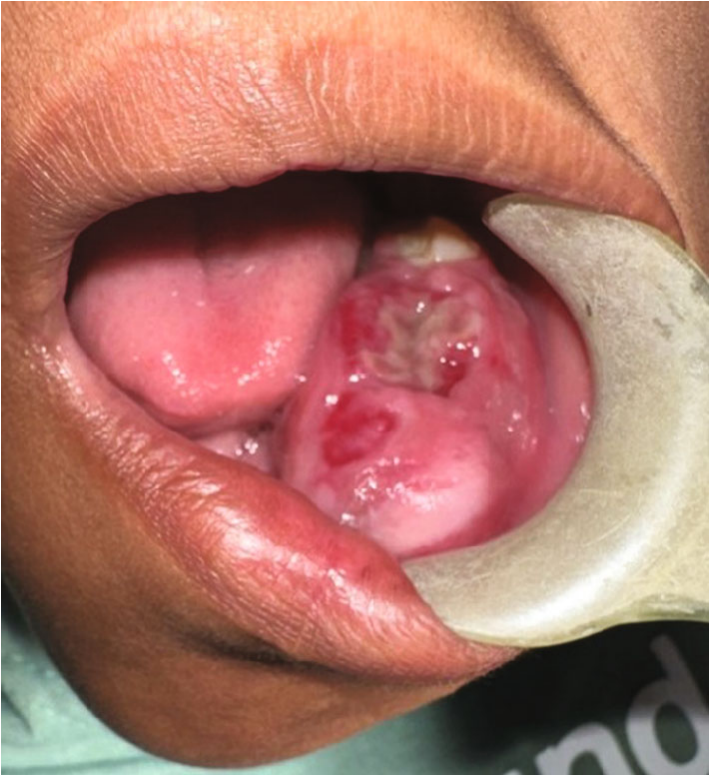
The expansile lesion in the 3rd quadrant with overlying mucosal ulceration.

In January 2024, a 12-year-old mixed-race female presented at the University of The Western Cape Oral Health Centre with a 2-month history of an extensive mass in the left mandible, resulting in notable facial asymmetry. The patient's medical history was unremarkable. No palpable lymph nodes were evident, but paraesthesia was present along the left inferior alveolar nerve. Intraorally, an irregular, nonpulsatile, expansive lesion with ulcerated areas was evident (Figure 1).

**Figure 2:**
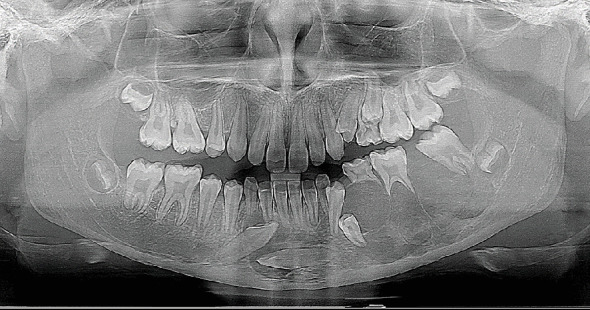
Panoramic radiograph showing the multiloculated lesion in the left body of the mandible. The “floating teeth” appearance can also be seen.

Panoramic imaging (Figure 2) revealed a partially defined, irregular, multilocular radiolucency with thin radiopaque septa in the third quadrant. The lesion extended medially, crossing the midline and encompassing the crowns of the impacted Teeth Nos. 33 and 43 (FDI classification). There was also root resorption of Tooth 36, displacement of both erupted and unerupted permanent teeth, and loss of lamina dura, giving the appearance of “floating teeth.”

**Figure 3:**
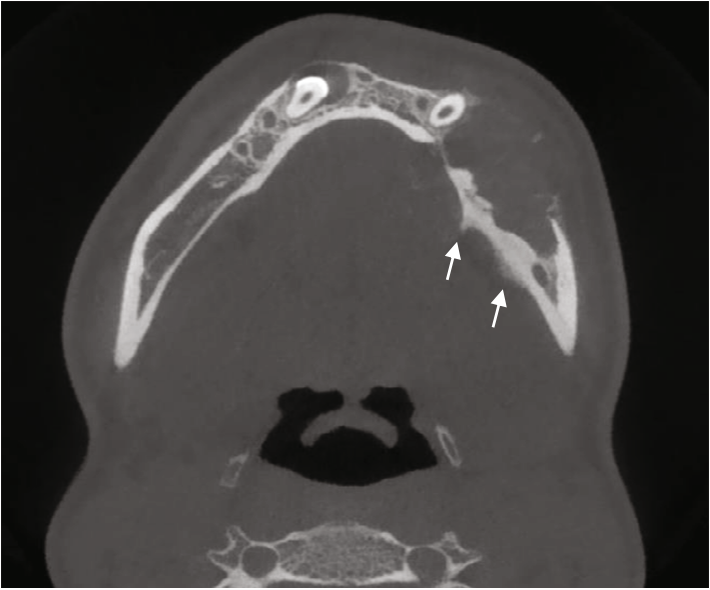
Axial CBCT slices showing a partially defined, expansile hypodensity extending across the midline to involve the impacted canines. Hyperdense bony spicules can be seen within the lesion, as well as Codman's triangle on the lingual aspect of the mandible (white arrows).

**Figure 4:**
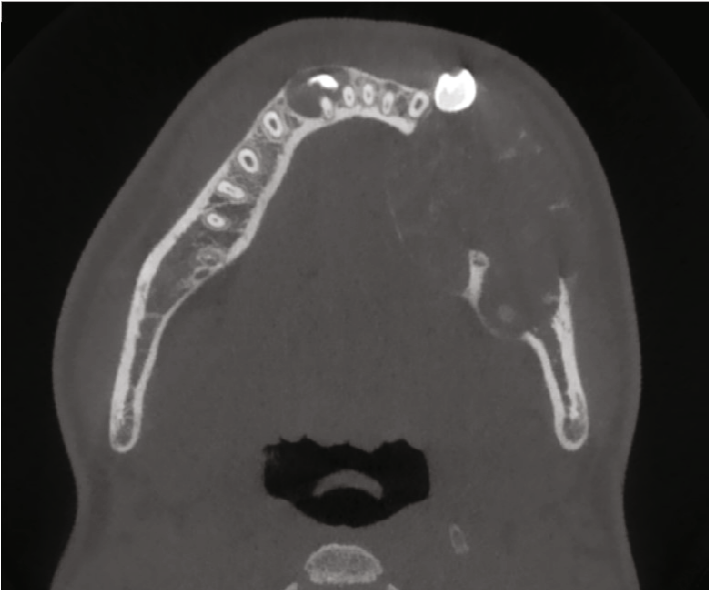
Axial CBCT slices showing a partially defined, expansile hypodensity extending across the midline to involve the impacted canines. Hyperdense bony spicules can be seen within the lesion.

**Figure 5:**
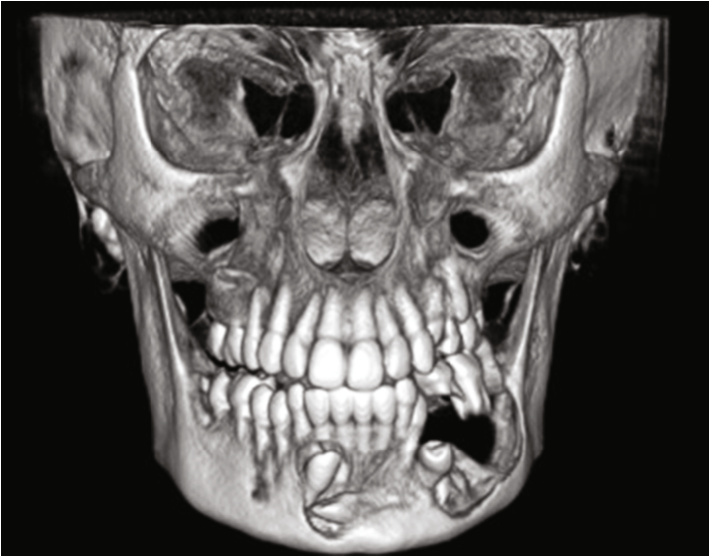
A 3D reconstruction of the lesion (frontal view).

**Figure 6:**
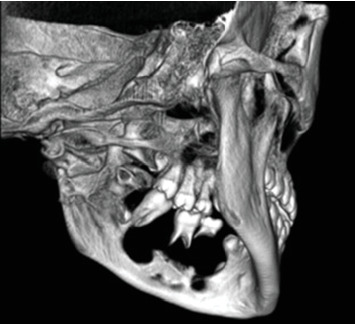
A 3D reconstruction of the lesion (posterior oblique view).

Cone-beam computed tomography (CBCT) imaging (Figures 3, 4, 5, and 6) showed a partially defined, expansile hypodensity containing hyperdense spicules in the body of the left mandible, extending from the angle to the right parasymphysis region, around the impacted canines. Codman's triangle was observed on the lingual aspect of the mandible, along with involvement of the inferior alveolar nerve on the left side. The differential diagnosis included osteosarcoma, leukaemia, lymphoma, haemangioma, and ameloblastoma.

Following the initial consultation, the patient had an incisional biopsy done under general anaesthesia. Dental extractions were performed concurrently (Teeth 74 and 36) and the lesion biopsied via the extraction sockets.

**Figure 7:**
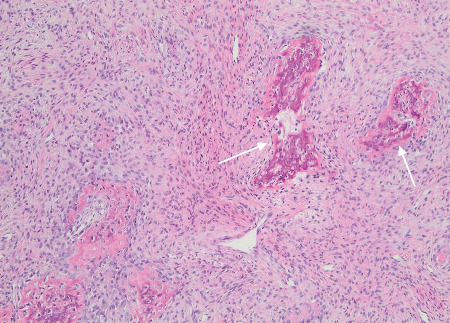
The tumour shows foci of malignant bone (osteoid; arrows; H&E, ×10).

**Figure 8:**
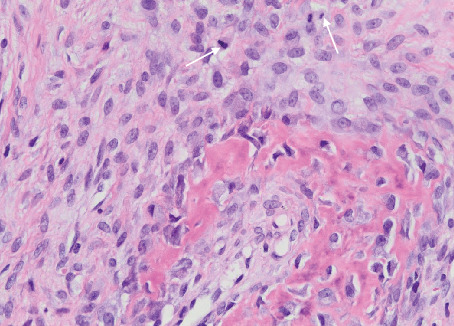
A focus of osteoid with entrapped atypical osteoblasts. There were two atypical mitotic figures in this high power field (arrows; H&E, ×40).

The biopsy revealed tumour tissue consisting of malignant osteoid with regions of mineralization (Figures 7 and 8). The tumour cells in between were spindled, exhibiting eosinophilic cytoplasm and irregular hyperchromatic nuclei. Scattered osteoclast-type giant cells were observed. Atypical mitotic figures were detected (arrows). This confirmed a diagnosis of conventional osteosarcoma.

**Figure 9:**
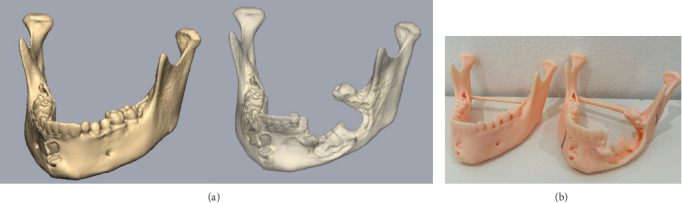
(a) Pre-surgical digital planning using CBCT reconstruction. (b) 3D model.

**Figure 10:**
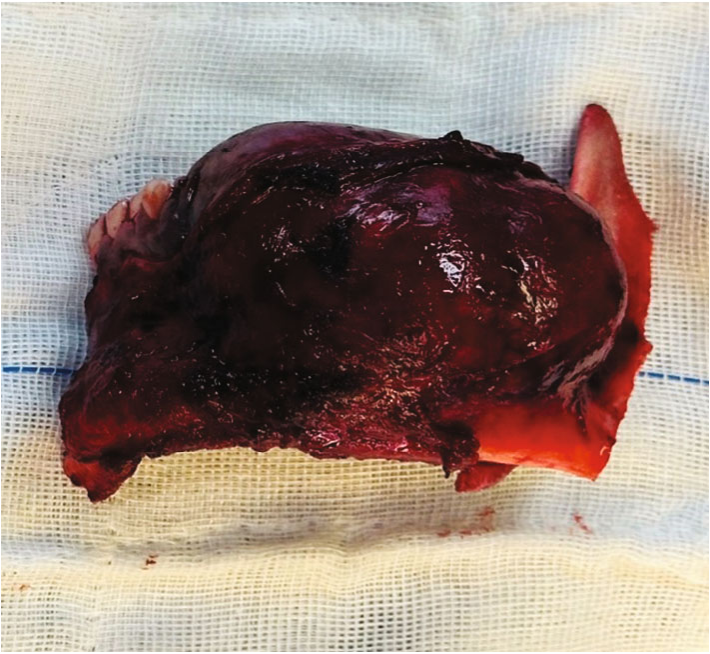
Resected tumour.

**Figure 11:**
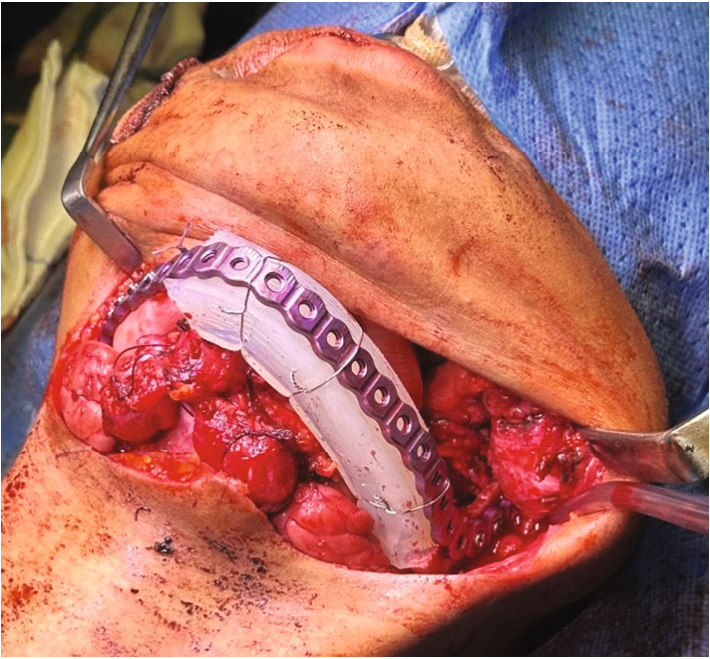
Temporary reconstruction with a titanium plate and silastic spacer.

After establishing a histopathological diagnosis, surgical preparation commenced, and the patients' CBCT scan was processed to generate a digital three-dimensional model. Tumour margins were then easily demarcated and excised digitally with the defect reconstructed by a mirror image of the unaffected contralateral side (Figure 9). A titanium reconstruction plate was then manually adapted to the three-dimensional model prior to surgery to re-establish the bony mandibular contour. A hemimandibulectomy with 10 mm clear surgical margins was performed (Figure 10) using extraoral approaches and the remaining mandibular segment fixated with the preadapted titanium reconstruction plate. A silastic spacer was secured to the titanium plate by means of wires (Figure 11) which would allow for the reconstruction of the defect using a particulate posterior iliac crest bone graft in the future. Bilateral Level I and Level II neck lymph nodes were also biopsied at the same time of surgery and demonstrated no features of malignancy. Three weeks after surgery, the patient was initiated on EURAMOS protocol of doxorubicin and cisplatin [13]. The patient has since completed the chemotherapy regimen and, as of February 2025, is no longer on chemotherapy. Currently, the patient is undergoing evaluation for a fibula free flap reconstruction to address the osseous defect.

## 3. Discussion

A search on PubMed and Google Scholar (“Osteosarcoma” [MeSH] AND “Jaw” [MeSH] AND “Case Reports” [Publication Type] AND “Child” [MeSH]) for case reports on OSJ in children aged 12 years and younger found 12 cases (in English) from accredited journals. These cases are summarized in Table 1.

While the precise cause of osteosarcoma is unknown, mutations in the RB and p53 genes significantly contribute to its onset. Individuals with RB germline mutations face a 1000-fold increased risk of developing osteosarcoma. Similarly, those with p53 germline mutations, known as Li–Fraumeni syndrome, also show a higher incidence of this tumour [24]. Notably, osteosarcoma is more likely to arise in areas of bone growth, probably due to the proliferation of osteoblasts, which are more prone to transformation [25].

OSJ can be classified into two types: primary and secondary. The aetiology of the primary type is unknown but may be associated with rapid bone growth during adolescence, environmental factors such as ultraviolet radiation, exposure to methylcholanthrene, chromium salts, beryllium oxide, asbestos, aniline dyes, and a genetic predisposition [8]. The secondary type occurs in older individuals with conditions like Paget's disease, fibrous dysplasia, chronic osteomyelitis, or as a late complication of radiation therapy to the craniofacial region [9]. In the case described by Goncalves et al. [23], the patient had a history of malignancy in the orofacial region, which was subsequently treated with chemo- and radiotherapy. Although this patient was a 12-year-old, the radiotherapy could have been the initiating factor for the development of secondary OSJ.

A significant radiographic feature of OSJ on conventional imaging is Garrington's sign, which is the symmetric widening of the periodontal ligament (PDL) due to tumour cell infiltration, often seen in the early stages [9]. This was a radiographic feature in three [11, 16, 19] of the cases we identified in the literature. Other radiographic features may include tooth displacement, interdental bone loss, loss of lamina dura, and peripheral reactive cortical bone formation [26]. Conventional osteosarcoma often displays a characteristic “sunburst” or “sunray” appearance on radiographs due to the formation of thin, irregular spicules of new bone extending from the lesion as the tumour invades the periosteum [27]. Although this appearance is classic for long bone osteosarcomas, it is not pathognomonic for OSJ [7, 28] but was seen in three of the cases we highlighted in the literature [11, 12, 20]. Other features may include Codman's triangle, resulting from the elevation of the bony cortex due to new periosteal bone formation over the expanding tumour. This was not seen in any of the 12 cases presented except for our case. However, radiographic features can vary significantly due to the destruction of normal bone and the degree of mineralization of the newly formed bone [10].

Computed tomography (CT) and magnetic resonance imaging (MRI) are useful for assessing the bone destruction pattern in OSJ, which can present as lytic, sclerotic, or mixed. The osteolytic variant appears completely radiolucent, while the mixed pattern shows radiopaque spicules within a radiolucent mass, and the osteosclerotic pattern shows high-density new bone formation [6]. From the information gathered on the 10 patients who had either a CT or MRI, the most common pattern encountered was the mixed variant [11, 12, 15, 17, 19, 20, 22], which coincided with our case.

Histopathologically, the diagnosis of osteosarcoma is based on identifying osteoid production by malignant mesenchymal cells, though this alone is insufficient. Osteosarcoma can be classified into central and peripheral subtypes. Central osteosarcoma histotypes may include conventional, high-grade central, low-grade central, epithelioid, and telangiectatic [1]. The main types of cellular differentiation in conventional osteosarcoma are osteoblastic, chondroblastic, and fibroblastic, with the final diagnosis depending on the type of extracellular matrix produced by the tumour cells [8]. Typically, one histologically predominant pattern is observed, although two patterns may be seen in some lesions [26]. Peripheral osteosarcoma histotypes may include parosteal (juxtacortical), low-grade, and high-grade, among others [1].

As per our review, two patients had epithelioid osteosarcoma [14, 18], two had telangiectatic osteosarcoma [15, 19], three patients had conventional osteosarcoma [11, 12, 22], one had high-grade osteosarcoma [23], two had low-grade osteosarcoma [20, 21], one had parosteal (juxtacortical) osteosarcoama [17], and one case was unspecified [16]. Our patient's diagnosis aligned with the most common type of osteosarcoma we found in the literature—the conventional type.

However, diagnosing OSJ can be complex on a histological level, often requiring multiple sequential biopsies to confirm the diagnosis [29]. Therefore, a combination of clinical, radiographic, and histopathologic features is needed to arrive at a definitive diagnosis.

Primary treatment for OSJ involves radical surgical removal with clear margins of 1.5–2 cm [4]. A hemimandibulectomy is usually the treatment of choice for mandibular OSJ, while complete resection is challenging for maxillary OSJ, leading to a higher likelihood of local recurrence [26]. Therefore, routine CT scans are important for maxillary OSJ patients to assess for soft tissue extension, particularly into the nasal cavity, antral floor, and floor of the orbit [5].

Chemotherapy, typically recommended before surgery for osteosarcomas in long bones, remains a point of debate for OSJ [9]. OSJ exhibits lower likelihood of distant metastasis, and patients generally achieve a 5-year survival rate of 77% if the disease is localized and fully resected [4, 9]. In our review, three patients [15, 18, 21], including our patient, received chemotherapy either before surgery, after surgery, or both and have shown no signs of recurrence for over a year, with one patient [21] remaining disease-free for 5 years. However, patients who did not receive chemotherapy and were not palliative or unspecified [12, 17, 20] also responded well to treatment, with one patient [17] being disease-free for 3.5 years.

Histologic subtypes have been found not to significantly impact prognosis. Therefore, regular follow-up is essential for all osteosarcoma variants due to their tendency to spread along the PDL, inferior alveolar nerve, mandibular foramen, mandibular canal, and marrow spaces when located in the jaw [5].

## 4. Conclusion

Since OSJ lacks definitive clinical and histopathological features, careful evaluation is essential for each case presenting with unusual radiographic characteristics. This emphasizes the importance of a thorough, multidisciplinary approach in diagnosing and managing such lesions. By integrating clinical observations, radiographic findings, and pathological analysis, healthcare professionals can achieve a more accurate diagnosis and develop an effective treatment plan. This comprehensive correlation is crucial for identifying the nature of the tumour and ensuring optimal patient outcomes.

## Figures and Tables

**Table 1:** A summary of case reports involving osteosarcoma of the jaw in children 12 years and younger.

**Authors, year of publication**	**Patient's age, sex**	**Anatomic site**	**Clinical findings**	**Radiological features**	**Histological subtype**	**Treatment**	**Follow-up**
Kaveri et al., 2009 [14]	4 years old, M	Maxilla	Swelling, medical/dental history not specified	CT: Hypodense lesion extending into nasal cavity, maxillary sinus, and orbital cavity. Destruction of the medial and lateral wall of the maxillary antrum, inferior orbital walls, and nasal septum	Epithelioid OS	Weber–Ferguson approach—excision of mass. Pt's parents refused any further treatment	Not specified
Jot et al., 2023 [15]	6 years old, M	Maxilla (anterior)	Swelling, history of trauma to the anterior maxilla by a wooden stick while playing	OPG: Well-defined radiolucency in the maxilla, involving the alveolar process CT: Heterogenous, intensely enhanced lesion with internal calcification foci in the midline of the maxilla	Telangiectatic OS	×3 cycles neoadjuvant chemotherapy followed by surgical resection and ×4 cycles of adjuvant chemotherapy	No evidence of recurrence 1 year, 3 months post-op
Miceli et al., 2020 [16]	7 years old, M	Mandible (body)	Pain, swelling, medical/dental history not specified	(Type of imaging not specified) Widening of the PDL of Tooth 73, alteration of the trabeculae in the 74 and 75 regions	Osteosarcoma, NOS	Marginal resection	No evidence of recurrence 2 years post-op
Donaldson et al., 2004 [17]	8 years old, M	Mandible (body)	Swelling. No significant medical/dental history	Occlusal radiograph: Irregular mixed lesion on the buccal aspect of the mandible CT: Nonhomogenous hyperdensity with ill-defined margins infiltrating the buccal cortex in the second premolar and first molar regions. Superficial extension of the lesion into the trabecular component of the mandible was also noted	Parosteal (juxtacortical) OS	Marginal resection of the mandible and surrounding soft tissue	No signs of recurrence 3.5 years post-op
Cozza et al., 2009 [18]	8 years old, F	Mandible	Swelling, fever, pain	CT: Hypodense lesion on the left mandible with soft tissue oedema	Epithelioid OS	Pre-op chemotherapy (×2 cycles) followed by a hemi-mandibulectomy and fibular flap reconstruction. Post-op adjuvant chemotherapy was administered (×13 cycles)	No signs of recurrence 42 months post-op
Tomar et al., 2016 [19]	9 years old, M	Mandible (body)	Swelling, pain, palpable submandibular and cervical lymph nodes. No significant medical/dental history	Periapical and OPG: Widening of the PDL with irregular attenuation of the lamina dura in relation to Teeth 74, 75, and 36. Disruption of follicular space and lining relating to Tooth 35 was evident Occlusal: Presence of fine radial spicules and mild cortical erosion in 75 region CT: Confirmed conventional radiological findings MRI: Patchy altered marrow in body of mandible with erosion of alveolar margins, multiple enlarged lymph nodes at Levels 1a, 1b, and 2 of the neck bilaterally	Telangiectatic OS	Not specified	Not specified
Bilodeau et al., 2010 [20]	9 years old, M	Mandible (body)	Pain, swelling, medical/dental history not specified	OPG: Well-defined radiopacity with thin peripheral, radiolucent halo around roots of Tooth 36. Radiopaque “sunburst” pattern seen emanating from root surface. Mandibular canal displaced inferiorly CT: Thinning of cortical plate secondary to buccal-lingual expansion. Surrounding soft tissues displayed oedema and reactive changes	Low-grade OS	Marginal resection with fibula graft	No signs of recurrence 2 years post-op
Kupeli et al., 2012 [21]	10 years old, F	Maxilla	Swelling, pain, medical/dental history not specified	CT: Ill-defined, expansive mass at left maxillary alveolar process, extending to the palate and maxillary sinus. Bone destruction present	Low-grade OS	Maxillectomy followed by chemotherapy (×6 cycles)	No signs of recurrence 5 years post-op
Nirmala et al., 2014 [11]	10 years old, F	Mandible (body)	Swelling. No significant medical/dental history	Periapical: Symmetrical widening of PDL space with altered bony trabecular pattern in 35 and 36 regions OPG: Well-defined radiolucency with patchy areas of radiopacity internally CT: Partially defined hypodensity with erosion of buccal cortical plate and “sunburst” appearance	Conventional OS (fibroblastic type)	Marginal mandibulectomy	Not specified
Yamamoto et al., 2011 [22]	11 years old, M	Maxilla (anterior)	Swelling, history of afebrile convulsions	OPG and occlusal: No abnormal findings noted CT: Partially defined hypodensity with bone destruction on hard palate extending into maxilla. Slightly heterogenous internal structure MRI: Ill-defined hypointensity with heterogenous internal structure	Conventional OS (fibroblastic type)	Not specified	Not specified
Goncalves et al., 2024 [23]	12 years old, F	Mandible	Pain, tooth mobility, history of parameningeal alveolar rhabdomyosarcoma at 2 years old—treated with chemo- and radiotherapy	Not specified	High-grade OS	Palliative	Pt succumbed to her illness 7 months after diagnosis
Madiraju, 2021 [12]	12 years old, F	Mandible (body)	Swelling. No significant medical/dental history	OPG: Erosive bony changes with adjacent soft tissue invasion CT: Expansile hypodensity with divergent bony spicules perpendicular to the underlying cortex (“sunburst” appearance) at the border of the angle of the mandible	Conventional OS (fibroblastic type)	Surgical resection	No signs of recurrence 6 months post-op

Abbreviations: CT = computerized tomography, F=female, M = male, MRI = magnetic resonance imaging, NOS = not otherwise specified, OPG = orthopantomogram, OS = osteosarcoma, PDL = periodontal ligament.

## Data Availability

The data supporting this case report are available from the corresponding author upon reasonable request.
